# Buccelli on Effects of Tobacco

**Published:** 1898-02

**Authors:** 


Buccelli (*Riv. di Patolog. Nerv.*, 1896, p. 327) concludes from his investigations of 200 patients with nerve and brain troubles, and others, that tobacco effects the normal nervous system to a comparatively trifling extent, but as soon as the condition of perfect integrity is impaired, its effect is extremely and progressively pernicious. The subcortical and bulbar nerve centers suffer particularly then from the toxic effect of tobacco.—*Medical and Surgical Reporter.*

Chicago physicians object to the use of cocaine on firemen's eyes as practiced in New York. They claim that cocaine thus used is apt to give rise to the habitual use of the drug.

